# Naringenin improves testicular reproductive function in rats with testicular ischemia-reperfusion injury

**DOI:** 10.1590/acb410426

**Published:** 2026-01-16

**Authors:** Si-Ming Wei, Yu-Min Huang

**Affiliations:** 1 Zhejiang Shuren University – Shulan International Medical College – Hangzhou, Zhejiang – China.; 2 Zhejiang Chinese Medical University – School of Nursing – Hangzhou, Zhejiang – China.; 3 Zhejiang University – College of Education – Department of Sports Science – Hangzhou, Zhejiang – China.

**Keywords:** Antioxidants, Testis, Spermatic Cord Torsion, Ischemia, Reperfusion Injury, Rats

## Abstract

**Purpose:**

The significant change during testicular ischemia-reperfusion is the generation of high levels of reactive oxygen species, which trigger impairment of spermatogenic cells. Naringenin, a plant-derived flavonoid, can alleviate oxidative stress. The current study was conducted to examine the possible protective ability of naringenin on testicular ischemia-reperfusion injury.

**Methods:**

Three groups of male rats were created: group 1 (sham operation), group 2 (left testicular ischemia-reperfusion), and group 3 (treatment with naringenin after left testicular ischemia-reperfusion). Testicular ischemia of rats was induced by 2 hours of left testicular torsion, and subsequently testicular detorsion was performed for reperfusion. Rat testes of three groups were taken to analyze nicotinamide adenine dinucleotide phosphate (NADPH) oxidase activity, which contributes to the production of reactive oxygen species, malondialdehyde content (an index of reactive oxygen species), and testicular reproductive function.

**Results:**

The NADPH oxidase activity and malondialdehyde content were higher in ipsilateral testes, but testicular reproductive function was lower in testicular ischemia-reperfusion group than in sham group. Conversely, NADPH oxidase activity and malondialdehyde content decreased in ipsilateral testes after naringenin treatment, leading to enhanced testicular reproductive function.

**Conclusion:**

Naringenin reduced NADPH oxidase activity and inhibited generation of reactive oxygen species to achieve protection of testicular reproductive function.

## Introduction

Testicular torsion is characterized by the spermatic cord rotation around its axis and ranks among the foremost emergencies in urology. It affects one per 4,000 males by 25 years^1^. After testicular torsion, testicular blood flow is interrupted, leading to testicular ischemia. Rapid diagnosis (within 6 hours) and surgical intervention (testicular detorsion) can resolve testicular ischemia and prevent infarction of the torsional testis^1^. Even when testicular detorsion is conducted immediately, 25–66.3% of patients still end up with testicular atrophy^2^^,^ 3. The injury to the testis results from oxidative stress^4^. The significant change during testicular ischemia-reperfusion is the generation of high levels of reactive oxygen species like hydroxyl radicals, superoxide anion, and hydrogen peroxide^4^. Reactive oxygen species can attack and react with cellular components like lipids, proteins, DNA and carbohydrates, triggering impairment of spermatogenic cells^4^.

Up to the present, no effective therapeutic agent has been used to fight against testicular ischemia-reperfusion injury in the clinic. Naringenin (4,5,7-trihydroxy-flavanone), a plant-derived flavonoid, widely exists in citrus fruits, such as orange, grapefruit, cherry and tomato^5^. Its molecular formula and molecular weight are C_15_H_12_O_5_ and 272.25, respectively^6^. A variety of pharmacological studies have confirmed that naringenin has remarkable properties of antioxidation, anti-inflammation, anti-microbe, anticancer, and so on^6^^,^ 7. It has been found that naringenin has positive effect in mitigating ischemia-reperfusion injury in vital organs, including the heart, pancreas, brain, kidney, skin flap, and retina^6^^–^ 11. Nevertheless, it remains unknown whether naringenin has a potential protective effect on testicular ischemia-reperfusion injury. Therefore, we aimed to evaluate the effect and mechanism of naringenin on ischemia-reperfusion damage after testicular torsion-detorsion.

## Methods

### Experimental animals and ethical approval

We acquired male Sprague-Dawley rats weighing 250–300 g from the SLAC Company (Shanghai, China). The animals were kept in polypropylene cages in controlled air-conditioned animal facilities with a humidity ranging from 50–60%, 12-hour periods of light and dark cycle, and a temperature ranging from 20–22ºC. They had unrestricted access to standard rat food and fresh water. The rats took seven days to become acclimatized to the controlled conditions prior to experiments.

The Zhejiang Chinese Medical University Ethics Committee approved all the protocols involving animals, with ethics approval code 10790. All animal experiments were achieved in strict adherence to the policies of Laboratory Animal Care and Use.

### Experimental groups and surgical procedure of testicular ischemia-reperfusion induction

Rats were randomly allocated to the following groups, with 20 rats in each group:

- Group 1: sham-operated group;
- Group 2: left testicular ischemia-reperfusion group;
- Group 3: left testicular ischemia-reperfusion group with naringenin treatment.

General anesthesia was induced with the help of 50 mg/kg ketamine (intraperitoneal; Sigma-Aldrich, Saint Louis, MO, United States of America). The fur over the skin was shaved in the left ilioinguinal and scrotal areas, and 10% povidone-iodine solution was used to cleanse the skin. The left testes of rats in the three groups were extracted via a left ilioinguinal incision. Rats in the sham group experienced placement of 11/0 noninvasive suture through the tunica albuginea, and then the testis was positioned into the scrotum without testicular torsion-detorsion. The incision was sutured with 4/0 silk thread.

In the testicular ischemia-reperfusion group, we twisted left testis 720 degrees in counterclockwise direction around the spermatic cord and fixed the rotated testis to the lateral scrotal wall for 2 hours using 11/0 noninvasive suture to prevent testicular spontaneous detorsion^12^. After testicular ischemia period, testicular reperfusion was conducted through counter-rotation of the twisted testis to its anatomically right position^12^. Naringenin (50 mg/kg, intraperitoneal, Sigma-Aldrich) was administered to rats in the naringenin-treated group at the beginning of testicular reperfusion. The dose of naringenin was based on several previous studies showing effectiveness of the dose in treating ischemia-reperfusion injury in various organs^6^^,^ 8 ^–^ 10.

The 10 rats from each experimental group were collected at the fourth hour following reperfusion. We obtained left and right testes of rats to evaluate activity of nicotinamide adenine dinucleotide phosphate (NADPH) oxidase, and malondialdehyde level. The remaining 10 rats from each experimental group were collected at the third month following reperfusion. The left and right testes of rats were harvested to assess testicular reproductive function.

### Determination of testicular NADPH oxidase activity

Fresh testicular specimen was homogenized on ice in lysis buffer and centrifuged at 10,000 × g for 10 minutes at 4°C. Then, the supernatant was collected to assess the activity of NADPH oxidase. A commercial kit (Jiancheng Bioengineering Institute, Nanjing, China) was utilized to detect NADPH oxidase activity.

### Testicular malondialdehyde activity assay

Testicular tissue was weighed 100 mg and homogenized rapidly on ice in 1 mL of malondialdehyde lysis buffer. The homogenized testicular tissue was centrifuged for 15 minutes at 5,000 × g by a 4°C centrifuge to harvest supernatant for the evaluation of malondialdehyde level. Measurement of malondialdehyde level was conducted by an assay kit (Jiancheng Bioengineering Institute, Nanjing, China) in strict accordance with the supplier’s recommendations.

### Examination of testicular reproductive function

The testicular weight, Johnsen’s testicle scoring system, number of germinal epithelial layer, and seminiferous tubule diameter were applied to analyze testicular reproductive function^13^. Rat testis was harvested and weighed. For histopathological examination, sample of testicular tissue was submerged in a solution of Bouin for 4 hours. Testicular dehydration step was conducted using graduated ethanol series. Then, the specimen was subjected to clearing the process using xylene and paraffin embedding after which paraffin-embedded tissue block was sectioned with the help of a microtome at 5-μm thickness. Section of testicular tissue was dewaxed with xylene, hydrated with graded alcohol solutions, and stained for routine histopathological evaluation using hematoxylin and eosin (Sigma-Aldrich). Under a light microscope (200 × magnification), a blinded pathologist assessed 20 randomly selected seminiferous tubules from each section.

The evaluation of testicular spermatogenesis was carried out according to Johnsen’s testicle 10-level scoring system^14^. A score of 1 signifies no epithelial cells within seminiferous tubule^14^. A score of 10 signifies regular and whole spermatogenesis with a great number of sperms, and an open lumen in seminiferous tubule^14^. The number of germinal epithelial layer was counted from basal membrane to lumen in seminiferous tubule. Evaluation of seminiferous tubule diameter was conducted using a microscope ocular with micrometer.

### Data analysis

All data from biochemistry and histopathology were evaluated with statistical software (GraphPad Prism 4, San Diego, CA, United States of America). The normality of data distribution was verified with the aid of Shapiro-Wilk’s test. Descriptive statistics were given as mean and standard deviation. For comparisons among three experimental groups, one-way analysis of variance was employed. The post-hoc comparisons were performed with the Student-Newman-Keuls’ test. Comparisons between ipsilateral and contralateral testes in group were subjected to a two-tailed Student’s t-test. The *p* < 0.05 was selected as a cutoff to indicate statistical significance.

## Results

### Naringenin reduced NADPH oxidase activity in testis with ischemia-reperfusion

**Figure 1:**
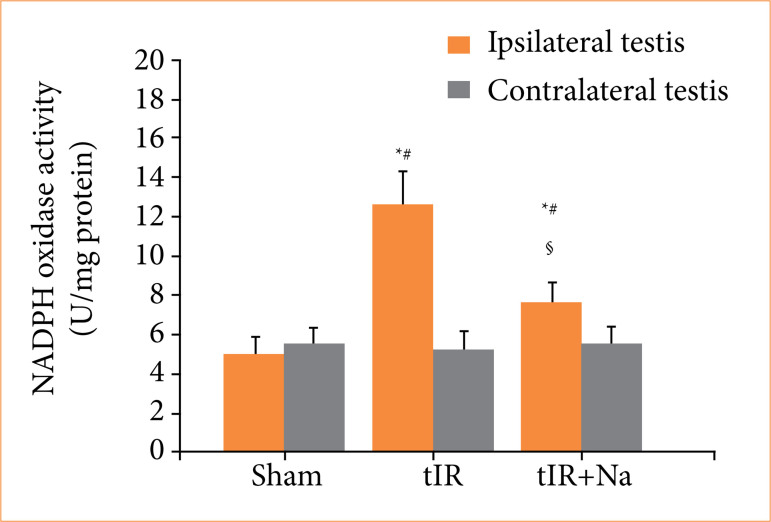
Naringenin (Na) treatment decreased ipsilateral testicular nicotinamide adenine dinucleotide phosphate (NADPH) oxidase activity. The NADPH oxidase assay was used to analyze NADPH oxidase activity in testes of sham-operated, testicular ischemia-reperfusion (tIR), and Na-treated experimental groups. Descriptive statistics were given as mean and standard deviation. The sample size was n = 10 independent observations.

During testicular ischemia-reperfusion, an increased activity of NADPH oxidase was observed in ipsilateral testis when compared to sham-operated group (*p* < 0.001; Fig. 1). The biochemical results showed a significant reduction in NADPH oxidase activity in ipsilateral testes subjected to treatment with naringenin in contrast to the testicular ischemia-reperfusion group (*p* < 0.001). A comparison of contralateral testes among three experimental groups showed no significant difference in NADPH oxidase activity (*p* > 0.05).

*Significant difference (*p* < 0.001) versus sham-operated group; #significant difference (*p* < 0.001) *versus* contralateral testes within group; §significant difference (*p* < 0.001) *versus* ipsilateral testes in tIR group. Source: Elaborated by the authors.

### Naringenin decreased malondialdehyde content in testis with ischemia-reperfusion

**Figure 2:**
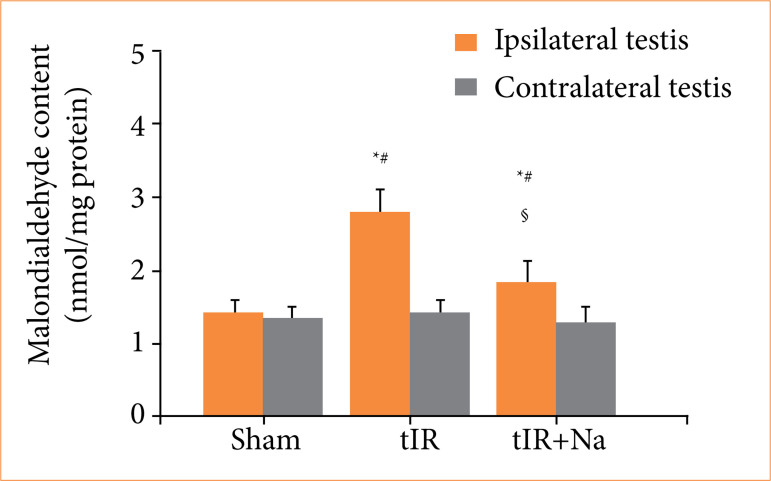
Naringenin (Na) treatment reduced ipsilateral testicular malondialdehyde content. Malondialdehyde assay was used to analyze malondialdehyde content in testes of sham-operated, testicular ischemia-reperfusion (tIR), and Na-treated experimental groups. Descriptive statistics were given as mean and standard deviation. The sample size was n = 10 independent observations.

As shown in Fig. 2, a comparison with sham-operated group revealed malondialdehyde content in ipsilateral testis to be increased in testicular ischemia-reperfusion group (*p* < 0.001). In addition, malondialdehyde content in ipsilateral testis was reduced in naringenin-treated group (*p* < 0.001 versus testicular ischemia-reperfusion group). In the statistical comparisons of contralateral testicular malondialdehyde content, it was observed that the difference among three experimental groups was not statistically significant (*p* > 0.05).

*Significant difference (*p* < 0.001) *versus* sham-operated group; ^#^significant difference (*p* < 0.0001) versus contralateral testes within group; ^§^significant difference (*p* < 0.001) *versus* ipsilateral testes in tIR group. Source: Elaborated by the authors.

### Naringenin promoted reproductive function in testis with ischemia-reperfusion

**Figure 3:**
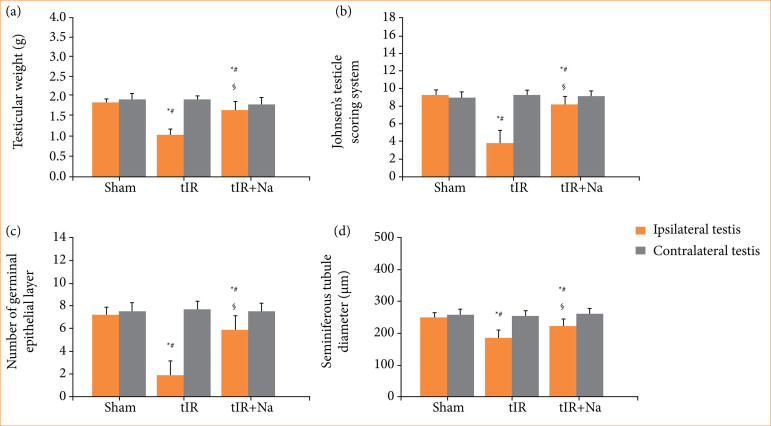
Naringenin (Na) treatment elevated ipsilateral testicular reproductive function, including **(a)** testicular weight, **(b)** Johnsen’s testicle scoring system, **(c)** number of germinal epithelial layer, and **(d)** seminiferous tubule diameter. Histopathological examination was used to analyze reproductive function in testes of sham-operated, testicular ischemia-reperfusion (tIR), and Na-treated experimental groups. Descriptive statistics were given as mean and standard deviation. The sample size was n = 10 independent observations.

**Figure 4:**
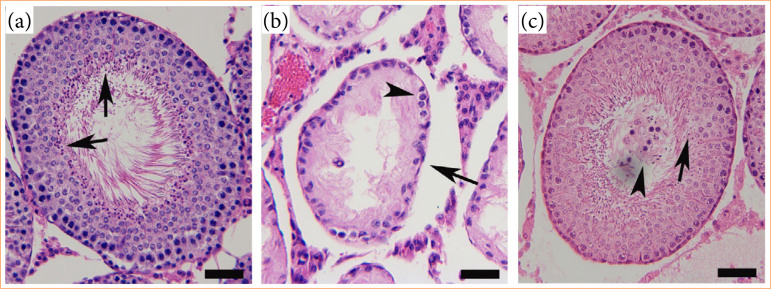
Typical microscopic findings of testicular sections stained with haematoxylin and eosin. **(a)** In ipsilateral testes of sham-operated group, and contralateral testes of sham-operated, testicular ischemia-reperfusion, and naringenin-treated groups, testicular seminiferous tubule displayed a great number of sperms (arrows), and well-arranged germinal epithelial layers. Furthermore, normal diameter of seminiferous tubule, and an open lumen in seminiferous tubule were also seen. **(b)** In ipsilateral testes of testicular ischemia-reperfusion group, testicular seminiferous tubule showed fewer germinal epithelial layers (arrowhead) and no sperms. Additionally, diameter of seminiferous tubule (arrow) was significantly atrophied. **(c)** In ipsilateral testes of naringenin-treated group, testicular seminiferous tubule was very close to sham-operated group in complete spermatogenesis with sperms (arrow), number of germinal epithelial layer, and seminiferous tubule diameter. Nonetheless, seminiferous tubular lumen was filled with exfoliated germinal epithelium (arrowhead). The seminiferous tubule is easily clogged by the exfoliated germinal epithelium (magnification: × 200; scale bar: 40 μm).

Source: Elaborated by the authors.

In ipsilateral testes, testicular reproductive function (such as testicular weight, Johnsen’s testicle scoring system, number of germinal epithelial layer, and seminiferous tubule diameter) was noted to decrease significantly (*p* < 0.001) in testicular ischemia-reperfusion group compared to sham-operated group (Figs. 3 and 4). Ipsilateral testes in naringenin-treated group showed higher reproductive function (*p* < 0.01) than those in testicular ischemia-reperfusion group. In sham-operated, testicular ischemia-reperfusion, and naringenin-treated groups, testicular reproductive function was found to be similar in contralateral testes (*p* > 0.05).

*Significant difference (*p* < 0.05) *versus* sham-operated group; #significant difference (*p* < 0.05) versus contralateral testes within group; §significant difference (*p* < 0.01) *versus* ipsilateral testes in tIR group. Source: Elaborated by the authors.

## Discussion

Once diagnosis of testicular torsion is made, prompt treatment (testicular detorsion) is vital to restore testicular blood flow and prevents testicular infarction. In clinical practice, the probability of testicular survival is 90–100% if patients receive testicular detorsion operation within 6 hours of testicular torsion (presenting with pain)^15^. The probability of testicular survival is 25–65% within 6–12 hours^15^. The probability drops to 0–25% within 12–24 hours^15^. Recent clinical studies revealed that 25–66.3% of torsional testes that were rescued developed testicular atrophy postoperatively^2^^,^ 3. In our study, after 2 hours of testicular torsion, ipsilateral testicular tissue damage, including marked reductions in testicular weight, Johnsen’s testicle scoring system, number of germinal epithelial layer, and seminiferous tubule diameter, was noted three months after testicular detorsion (Figs. 3 and 4).

The formation of reactive oxygen species is boosted after testicular torsion-detorsion^16^. Reactive oxygen species can modify cellular lipids, carbohydrates, DNA, and proteins, thereby contributing to spermatogenic cellular injury^4^. The content of reactive oxygen species is very difficult to determine directly in testicular tissue, because they have extremely high reactivity and short lifetime^12^. Reactive oxygen species cause cellular membrane damage by inducing the membrane lipid peroxidation^17^.

Malondialdehyde is a main secondary product during lipid peroxidation in cells^18^. Overproduction of reactive oxygen species during testicular torsion-detorsion leads to an increase in malondialdehyde content in cells^12^. Consequently, malondialdehyde content is commonly known as an index of reactive oxygen species^19^.

In the current study, testicular ischemia-reperfusion increased malondialdehyde concentration in ipsilateral testes, accompanied by a decrease in testicular reproductive function (Figs. 2–4). These results revealed that testicular reperfusion after ischemia leads to an accumulation of reactive oxygen species, which impairs spermatogenesis. However, malondialdehyde concentration showed notable reduction in ipsilateral testes of naringenin-treated rats, while there was an increase in testicular reproductive function when compared to testicular ischemia-reperfusion rats (Figs. 2–4). Our findings exhibited that application of naringenin after testicular ischemia-reperfusion ameliorates testicular reproductive function through decreasing oxidative stress. Some clinical studies have proved that naringenin is beneficial and safe in treating non-alcoholic fatty liver disease, bronchial pneumonia, and so forth^20^^,^ 21. Therefore, naringenin may be proposed as a promising drug for clinical therapy of testicular ischemia-reperfusion injury. However, the mechanism via which naringenin decreases oxidative stress remains to be elucidated.

The NADPH oxidase in neutrophil contributes to the production of reactive oxygen species in ischemia-reperfusion tissue^22^. At ischemia stage, calcium ions enter in neutrophil and cause activation of intracellular NADPH oxidase^23^. At the reperfusion stage, recovery of blood flow brings oxygen to ischemic tissue^24^. The NADPH oxidase reduces oxygen to superoxide anion^25^. Hydrogen peroxide is generated via the reaction of superoxide anion with itself^26^. Hydroxyl radical is produced through the reaction between hydrogen peroxide and superoxide anion^26^. Therefore, reperfusion of ischemic tissue results in release of reactive oxygen species.

In the current study, NADPH oxidase activity and malondialdehyde concentration in ipsilateral testes of testicular ischemia-reperfusion group showed a remarkable increase, while testicular reproductive function reduced significantly (Figs. 1–4). These data indicate that NADPH oxidase activity is elevated through testicular ischemia-reperfusion process, and the high NADPH oxidase activity increases reactive oxygen species production, which can finally damage testicular reproductive function. However, naringenin-treated group showed a significant reduction in ipsilateral testicular NADPH oxidase activity and malondialdehyde concentration, whereas testicular reproductive function increased in contrast to testicular ischemia-reperfusion group (Figs. 1–4). These results suggested that naringenin provides protection in testicular ischemia-reperfusion injury via decreasing NADPH oxidase activity and reactive oxygen species level.

Whether unilateral testicular ischemia-reperfusion damages the contralateral testis is currently being disputed. Some studies presented that unilateral testicular ischemia-reperfusion disturbed contralateral testis and led to reduced testicular reproductive function^27^^,^ 28. However, some studies declared that no histopathological changes occurred in contralateral testis^29^^,^ 30. The present study revealed that there was no significant difference in contralateral testicular NADPH oxidase activity, malondialdehyde concentration, and reproductive function between sham-operated and testicular ischemia-reperfusion groups (Figs. 1–4). Hence, we consider that contralateral testis is not affected after induction of ischemia-reperfusion in ipsilateral testis.

There is a consensus that a causative link exists between inflammation and testicular ischemia-reperfusion injury^31^. The testicular response to ischemia-reperfusion involves substantial generation of pro-inflammatory mediators, including tumor necrosis factor-alpha (TNF-α) and interleukin-1beta (IL-1β), by both germ cells and resident macrophages in the interstitial tissue^32^^,^ 33. The chemotaxis of neutrophils into the testis is driven by these pro-inflammatory mediator signals during ischemia-reperfusion^32^^,^ 33. Neutrophils generate copious amounts of reactive oxygen species via their intracellular NADPH oxidase, inflicting damage upon the testis^31^^,^ 34. Our research elucidates that the protective mechanism of naringenin against testicular ischemia-reperfusion injury involves the suppression of NADPH oxidase activity and a reduction in reactive oxygen species. In our study, no investigation was conducted regarding the effects of naringenin on pro-inflammatory mediators and the influx of neutrophils into the testis. Future research will focus on examining how naringenin influences these factors.

## Conclusion

Our results established for the first time that naringenin decreases adverse effect of testicular ischemia-reperfusion on testicular reproductive function through lowering NADPH oxidase activity to inhibit generation of reactive oxygen species. These outcomes implied that naringenin holds potential as a valuable therapeutic agent to improve ischemia-reperfusion injury to testis in clinical practice. Therefore, we will aim to evaluate the clinical efficacy of naringenin in future human trials.

## Data Availability

The data will be available upon request.
